# Rapidly progressive dementia due to neurosarcoidosis

**DOI:** 10.1590/S1980-57642013DN74000012

**Published:** 2013

**Authors:** Gabriela Carneiro C. Fortes, Marcos Castello B. Oliveira, Laura Cardia G. Lopes, Camila S. Tomikawa, Leandro T. Lucato, Luiz Henrique M. Castro, Ricardo Nitrini

**Affiliations:** 1 Department of Neurology, Faculdade de Medicina da Universidade de São Paulo, São Paulo SP, Brazil.; 2 Department of Pathology, Faculdade de Medicina da Universidade de São Paulo, São Paulo SP, Brazil.; 3 Department of Radiology, Faculdade de Medicina da Universidade de São Paulo, São Paulo SP, Brazil.

**Keywords:** neurosarcoidosis, rapidly progressive dementia, diencephalic amnesia, primary CNS vasculitis

## Abstract

Rapidly progressive dementia (RPD) is typically defined as a cognitive decline progressing to severe impairment in less than 1-2 years, typically within weeks or months. Accurate and prompt diagnosis is important because many conditions causing RPD are treatable. Neurosarcoidosis is often cited as an unusual reversible cause of RPD.

**METHODS:**

We report two cases of neurosarcoidosis presenting as RPD.

**RESULTS:**

Case 1: A 61-year-old woman developed a RPD associated with visual loss. In seven months she was dependent for self-care. Magnetic resonance imaging (MRI) revealed temporal and suprasellar brain lesions. Treatment with high-dose intravenous prednisolone was associated with partial improvement. Case 2: A 43-year-old woman who was being treated for diabetes insipidus developed a severe episodic amnesia one year after onset of cognitive symptoms. Previous MRI had shown a hypothalamic lesion and she had been treated with oral prednisone and cyclophosphamide. There was reduction of the MRI findings but no improvement in the cognitive deficits. Brain biopsy disclosed noncaseous granulomas and granulomatous angiitis; treatment was changed to high-dose intravenous methylprednisolone, with poor symptomatic response.

**CONCLUSION:**

The diagnosis of RPD due to neurosarcoidosis can be challenging when the disease is restricted to the nervous system. In these cases, clinical presentation of RPD associated with neuroendocrine and visual dysfunction, imaging findings showing hypothalamic lesions and, in some cases, brain biopsy, are the key to a correct diagnosis. It is possible that earlier diagnoses and treatment could have led to a better outcome in these patients.

## Abstract (pt)

Demência rapidamente progressiva (DRP) é tipicamente definida como um declínio cognitivo que progride para prejuízo funcional severo em menos de 1-2 anos, geralmente em semanas a meses. O diagnóstico rápido e acurado é fundamental, já que muitas condições que levam a DRP são reversíveis.

**MÉTODOS:**

Relatamos dois casos de neurosarcoidose que se apresentaram como DRP.

**RESULTADOS:**

Caso 1: Uma mulher de 61 anos desenvolveu uma DRP associada a perda de acuidade visual. Em sete meses evoluiu com dependência para auto-cuidado. A ressonância magnética (RM) revelou lesões encefálicas temporais e supraselares. Evoluiu com melhora parcial após tratamento com metilprednisolona intravenosa em altas doses. Caso 2: Uma mulher de 43 anos que estava em tratamento para diabetes insipidus desenvolveu uma amnésia episódica severa um ano após o início dos sintomas cognitivos. A RM anterior mostrava uma lesão hipotalâmica, e ela recebeu tratamento oral com prednisona e ciclofosfamida. Houve redução dos achados da RM, porem sem melhora dos déficits cognitivos. A biópsia cerebral mostrou granulomas não caseosos e angeíte granulomatosa; o tratamento foi modificado para metilprednisolona intravenosa em altas doses, com resposta sintomática pobre.

**CONCLUSÃO:**

O diagnóstico de DRP por neurosarcoidose pode ser desafiador quando a doença está restrita ao sistema nervoso central. Nestes casos, a apresentação clínica da DRP associada a disfunção neuroendócrina e visual, exames de imagem com lesões hipotalâmicas e, em alguns casos, a biópsia cerebral são fundamentais para um diagnóstico correto. é possível que o diagnóstico e tratamento precoces poderiam ter trazido melhores resultados nesses pacientes.

## INTRODUCTION

Rapidly progressive dementia (RPD) can be defined as a dementing condition that progresses within 1-2 years, but occurs most commonly within weeks or months.^1^ The recognition of this entity is of great importance, since many of these disorders are potentially treatable, in contrast with the more common and irreversible slow-progressing neurodegenerative disorders, of which Alzheimer disease is the most prevalent.^2^

In a cohort of 825 patients with RPD referred with the presumptive diagnosis of Creutzfeldt-Jacob disease, Geschwind et al. found a 54% prevalence of prion disease, 28% of undetermined etiology (because of insufficient data), and 18% of other non-prion conditions, many of them treatable.^2^ Among these, 26% were neurodegenerative, 15% autoimmune, 11% infectious, 11% psychiatric, and 9% miscellaneous other, whereas 28% remained undetermined.^2^ Neurosarcoidosis is often cited as a cause of RPD, and its frequency is estimated at about 1.5% of non-prion RPD.^3^

Sarcoidosis can mimic many neurological conditions, and the clinical syndromes and brain magnetic resonance imaging (MRI) findings are highly variable. Symptomatic neurological manifestations are seen in approximately 5% of sarcoidosis patients.^4^ Approximately 28% of neurosarcoidosis patients have neurological symptoms as the initial manifestation. Of these, around 35% have isolated neurologic involvement.^4^ Neurosarcoidosis without clinical evidence of extraneural disease occurs in less than 1% of sarcoidosis patients.^4^ Cognitive changes occur in up to 26% of patients with neurosarcoidosis, and several cases presenting as RPD have been reported.^5-13^

Such patients represent a diagnostic challenge, since diagnostic criteria for neurosarcoidosis are frequently not fulfilled and alternative diagnoses often difficult to exclude.^14^ We present one case of probable and one of definite neurosarcoidosis, and discuss the difficulties regarding the diagnostic assessment and exclusion of other diseases.

## METHODS

We present two cases of RPD due to neurosarcoidosis, one case with probable and one with definite neurosarcoidosis, to review the diagnostic challenges of this rare cause of RPD.

**Case 1.** A 61-year-old woman developed social withdrawal, depressive mood, emotional lability and anhedonia 7 months before the diagnosis. She did not have any previous history of depression or psychiatric symptoms. Antidepressants were prescribed, without clinical improvement. Two months later, the patient manifested progressive bilateral visual loss, worse in the right eye. After one month, she developed memory difficulties for day-to-day events (i.e. forgetting that she had already had a meal, or forgetting to turn off the oven). Later on, she was unable to recall having attended her niece's wedding six months earlier. Long-term memory appeared intact. She began to present difficulties performing everyday tasks such as household chores, and, eight months later became progressively dependent for self-care.

**Figure 1:**
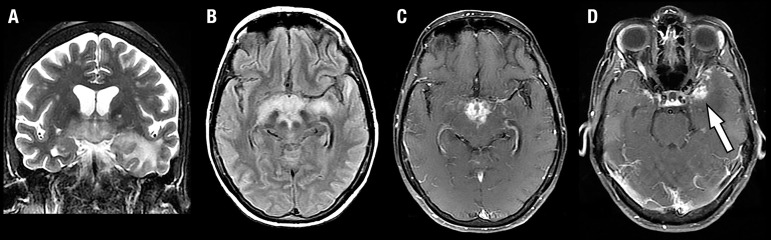
Case 1: Initial MRI. Coronal T2-weighted [A], axial FLAIR [B], and axial contrast-enhanced T1-weighted images [C and D] demonstrate extensive involvement of the hypothalamic and suprasellar regions, extending laterally towards the optic tracts, and to the left temporal lobe. The enhancing portion of the lesion is hypothalamic and suprasellar, and there is also a component of enhancement in the anterior portion of the temporal lobe (arrow in D).

On examination at our service, she was apathetic, exhibited a regular general state, with a blood pressure of 120x80 mmHg. Neurological examination disclosed severe attention deficit, her score on the Mini-Mental State Exam (MMSE) was 13/30 (she lost 5 points in temporal orientation, 3 in spatial orientation, 5 in attention/calculation, 3 in delayed recall, and 1 in the drawing of pentagons); she was unable to recite the months in reverse order and had a dysexecutive pattern on the clock drawing (4 points); her immediate memory was poor but she had even greater loss in delayed recall of the items with numerous intrusions; she had low semantic and phonemic fluency (12 animals and 7 letter p words in one minute) (Table 1). She had bilateral optic nerve involvement (visual acuity: counting fingers on right eye, 20/200 on left eye), with atrophy of the right papilla; gait, strength, reflexes, coordination and the remaining cranial nerves were preserved. Brain magnetic resonance imaging (MRI) showed hypothalamic, suprasellar, chiasmatic and optic tract lesions with hypersignal on T2 and FLAIR and contrast enhancement that extended to the diencephalic-mesencephalic transition, globus pallidus and left temporal lobe (Figure 1). Cerebrospinal fluid analysis showed mild pleocytosis (13 cells/mm^3^), slightly increased protein levels (67 mg/dl), and normal glucose levels. Erythrocyte sedimentation rate was 48 mm, and C-reactive protein was 100.

**Table 1:** Neurologic exam.

	Case 1	Case 2
Mood	Apathetic	Emotional lability
MMSE	13/30	13/30
Digit span (direct/indirect)	5 / 3	5 / 3
Short Memory Test with 10 items (incidental memory / immediate memory / learning)*	2 (2) / 5 (1) / 4 (2)	3 / 7 / 4
Delayed Memory Test with 10 items (after distraction) without / with hints*	0 (1) / 3 (1)	1 (1) / 6
Verbal fluency (semantic / phonemic)	12 (animals) / 7 (letter p)	5 (animals) / 3 (letter p)
Clock-drawing test	Dysexecutive (4 points)	Mild disturbance of pointers (8 points)
Somatic neurologic exam	Visual acuity: RE CF / LE 20/200	Normal

MMSE: Mini-Mental State Exam; RE: right eye; LE: left eye; CF: counting fingers.

* Number of intrusions in brackets.

Ê showed a slightly increased number of lymph nodes in the superior mediastinal, paratracheal, periaortic, subcarinal and cardiophrenic groups, measuring up to 15 mm. Hypothalamic-pituitary hormonal levels were normal.

She underwent a transbronchic lung biopsy that was nondiagnostic, revealing nonspecific inflammatory changes. One week later the patient presented decreased alertness, worsened apathy and muttered incomprehensible sounds, and responded only to simple commands. A brain biopsy was considered but as patient's condition was rapidly deteriorating, high-dose intravenous prednisolone was initiated, with prompt and significant improvement in consciousness level and self-care ability. Amnesic symptoms persisted. The patient was maintained on oral medication.

**Figure 2:**
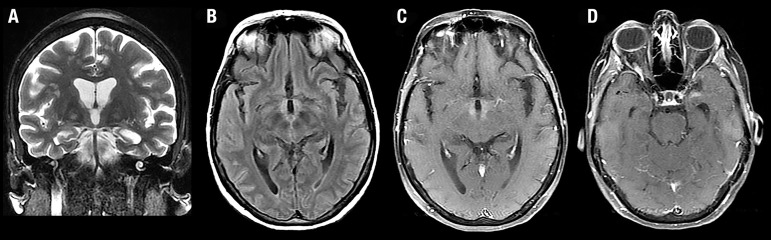
Case 1: Follow-up MRI. Coronal T2-weighted [A], axial FLAIR [B], and axial contrast-enhanced T1-weighted images [C and D] demonstrate almost complete resolution of the findings.

A repeat MRI, one month later, showed improvement of the previously encountered brain lesions (Figure 2). Systemic investigation showed no extraneural organ involvement. She was reevaluated six months later. Her husband reported inadequate behaviour and hyperphagia, and her MMSE was 13/30 (she lost 4 points in temporal orientation, 3 in spatial orientation, 4 in attention/calculation, 3 in delayed recall, 1 on verbal command, 1 on written command, and 1 in the drawing of pentagons); the remaining cognitive tests showed no significant improvement in memory or executive functions, compared with the initial assessment. Cerebrospinal fluid analysis was repeated, with 6 cells/mm^3^ and protein of 50 mg/dL. She was maintained on 20 mg prednisone a day and 10 mg methotrexate per week, and a monthly pulse therapy scheme was scheduled.

**Figure 3:**
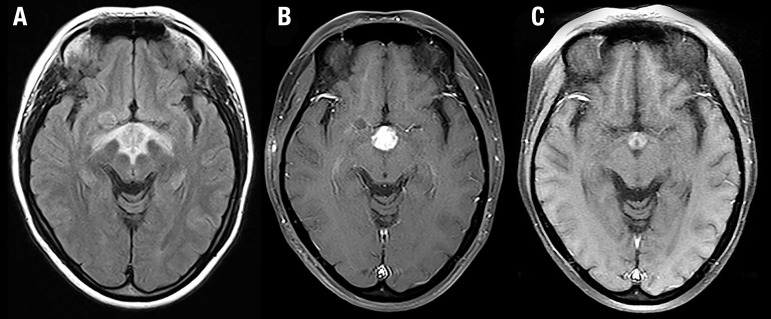
Case 2. Initial MRI. Axial FLAIR [A] and axial contrast-enhanced T1-weighted image [B] demonstrate extensive involvement of the hypothalamic and suprasellar regions, extending laterally towards the optic tracts. The enhancing portion of the lesion is hypothalamic and suprasellar. There is significant decrease in the size of the enhancing portion of the lesion in a follow-up axial contrast-enhanced T1-weighted image [C].

**Figure 4:**
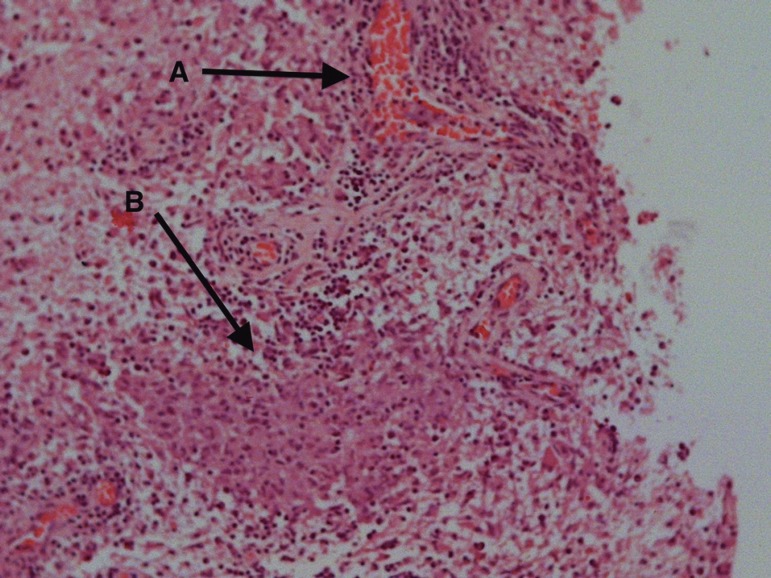
Case 2. Brain biopsy. Hypothalamic tissue showing perivascular mononuclear infiltrate [A] and interstitial non-caseous granulomas [B], consistent with sarcoidosis.

**Case 2.** A 43-year-old woman was diagnosed with diabetes insipidus four years earlier and treated with desmopressin. Medical records from that period were unavailable. The patient denied having used other medications. One year prior to presenting at our service, episodic memory dysfunction ensued. Memory difficulties worsened in a continuous and insidious manner, impacting daily activities. A stereotactic brain biopsy was performed four months later and was inconclusive. Brain MRI showed a contrast-enhancing expansive infiltrating hypothalamic lesion (Figure 3A and 3B). Chest and abdomen computed tomography were unremarkable. Brain biopsy was repeated one month later, and disclosed noncaseous granulomas and granulomatous angiitis of hypothalamic tissue (Figure 4). Ziehl Neelsen staining was performed, and Tuberculosis was excluded. The patient was treated with oral prednisone and cyclophosphamide.

On clinical evaluation at our service, after six months of treatment, the family reported persistent cognitive difficulties; she was dependent for most daily activities. The patient was still dependent on desmopressin for serum sodium control. Neurologic exam disclosed a score of 13 on the MMSE (she lost 5 points in temporal orientation, 4 in spatial orientation, 5 in attention/calculation, and 3 in delayed recall). Attention tests were altered, with a borderline digit span (direct: 5; indirect: 3). Memory tests revealed prominent episodic memory loss suggesting diencephalic involvement, with a delayed recall score of 1 out of 10 items, associated with one intrusion, and recall of 6 items after hints. Verbal fluency scores in one minute were 5 animals and only 3 words starting with the letter p (Table 1). Significant mood lability and confabulation was remarkable. Praxias, visuoconstructional and language domains were preserved. On the clock drawing test, she had a mild disturbance in pointers (8 points). She had normal muscle strength, reflexes were exalted on upper limbs and normal and symmetric on inferior limbs, a flexor plantar response, while coordination, sensitivity and cranial nerves were normal.

**Figure 5:**
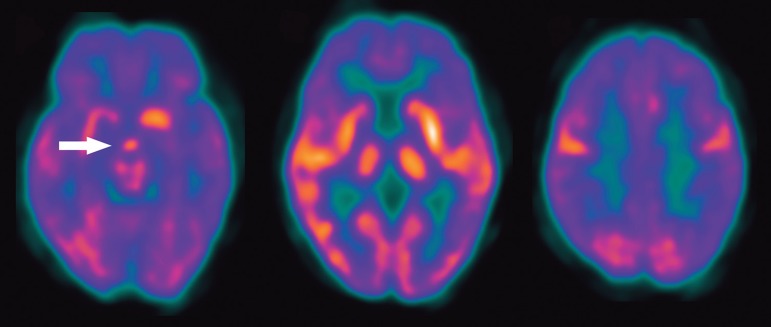
Case 2. PET [A-C]. An increase in glucose metabolism in the lesion is evident (arrow in A). Images at the level of the basal ganglia [B] and in the high convexity [C] show multiple areas of decreased metabolism throughout both cerebral hemispheres

A repeat MRI showed significant remission of the previously encountered lesion (Figure 3C). A head PET-CT showed increased glucose metabolism in the remaining lesion, but also demonstrated a widespread decreased glucose metabolism in the frontal, temporal and parietal lobes, bilaterally (Figure 5). Chest and abdomen CT were repeated, with the finding of parenchymal liver involvement (liver was not biopsied). Laboratory tests showed pan-hypopituitarism and slight increase in liver enzymes. She underwent high-dose intravenous methylprednisolone therapy, with poor symptomatic response. Four months later she was admitted to another hospital and died; we were unable to ascertain the cause of death or whether a necropsy was performed.

## RESULTS

In both cases, clinical and imaging findings were strongly suggestive of neurosarcoidosis. Also, both cases had cognitive or behavioral disturbances heralding nervous system involvement. In case 1, the condition manifested initially with depression and apathy, in a 61-year-old patient with no prior psychiatric history. Vision involvement rapidly ensued, followed by memory and executive function impairments, such that eight months after onset she was incapable of self-care. MRI showed contrast-enhancing lesions involving the left temporal lobe, left globus pallidus, hypothalamus and diencephalic-mesencephalic transition, besides the optic chiasm and optic tracts. Optic nerve and chiasm involvement are clinical manifestations that suggested the diagnosis of neurosarcoidosis.

In case 2, memory impairment was the predominant feature on cognitive exam with significant mood instability progressing to severe impairment in less than one year. MRI revealed contrast-enhancing lesions involving the hypothalamus. Endocrine dysfunction suggested neurosarcoidosis, but neoplastic disorders could not be excluded at first; brain biopsy was crucial for definite diagnosis. The diagnosis of neurosarcoidosis was challenging in both cases.

## DISCUSSION

In these two cases, cognitive manifestations were predominant, where both cases fulfilled clinical criteria for dementia,^15^ with the characteristics of diencephalic amnesia and rapidly progressive dementia.^1,2^

Current diagnostic criteria for neurosarcoidosis are still not validated or widely accepted.^4^ Diagnostic criteria proposed by Zajicek et al.^16^ require CNS tissue involvement (i.e. positive brain biopsy) for the definite diagnosis of neurosarcoidosis. Probable and possible neurosarcoidosis can be diagnosed based on typical clinical findings when biopsy is not available. Another set of diagnostic criteria^17^ requires demonstration of granulomatous inflammation in any organ, and additional neurologic clinical criteria, if extraneural tissue is biopsied. All diagnostic criteria require exclusion of diseases with similar manifestations. (Table 2)

**Table 2:** Diagnostic criteria for Neurosarcoidosis.

**Proposed diagnostic criteria of neurosarcoidosis by Zajicek et al.**	
Definite	Clinical presentation suggestive of neurosarcoidosis with exclusion of other possible diagnoses and the presence of positive nervoussystem histology
Probable	Clinical syndrome suggestive of neurosarcoidosis with laboratory support for CNS inflammation (elevated levels of CSF protein and/or cells, the presence of oligoclonal bands and/or magnetic resonance imaging (MRI) evidence compatible with neurosarcoidosis) andexclusion of alternative diagnoses together with evidence for systemic sarcoidosis (either through positive histology, including Kveimtest, and/or at least two indirect indicators from Gallium scan, chest imaging and serum ACE)
Possible	Clinical presentation suggestive of neurosarcoidosis with exclusion of alternative diagnoses where the above criteria are not met
Adapted from the proposed diagnostic criteria of neurosarcoidosis by Judson, et al.*	
Definite	• Positive MRI with uptake in meninges or brain stem
	• Cerebrospinal fluid with increased lymphocytes and/or protein
	• Diabetes insipidus
	• Bell’s palsy
	• Cranial nerve dysfunction
	• Biopsy of neural tissue showing granulomatous inflammation
Probable	• Other abnormalities on MRI
	• Unexplained neuropathy
	• Positive electrodiagnostic studies
Possible	• Unexplained headaches
	• Radiculopathy
• Assumes no other identified cause (such as infection, trauma, pre-existing condition, or co-existing disease) for the neurologic manifestation	
• Requires a tissue biopsy showing granulomatous inflammation in at least one extraneural organ unless the nervous system is biopsied.	

* Additional neurologic clinical criteria when an extraneural tissue is biopsied. A typical histopathologic finding on neural tissue alone defines the diagnosis of definite neurosarcoidosis.

Case one can be classified as probable neurosarcoidosis and case two as definite neurosarcoidosis, according to Zajicek et al's proposed criteria.^16^ Nearly all differential diagnoses, such as neurotuberculosis, systemic autoimmune disorders and brain neoplasm were excluded. In both cases, it was not possible to confirm extraneural disease: case one had mediastinal lymph nodes slightly increased in size and case two presented parenchymal liver involvement. These findings are compatible, but not specific, for systemic sarcoidosis.

Primary CNS angiitis (PCNSV) is more difficult to exclude in this setting, and can also present as a RPD.^18^ Neurosarcoidosis may show exquisitely angiocentric non-caseous granulomas and vascular wall damage, mimicking PCNSV.^19^ Brain biopsy features in case two, although suggestive of sarcoidosis because of the finding of interstitial non-caseous granulomas, also showed perivascular granulomas, which could also be consistent with the diagnosis of primary CNS granulomatous angiitis. In these cases, we must rely on clinical disease presentation. Although altered cognition is present in up to 53% of PCNSV cases, amnesic syndromes are rarely seen (9% of cases) in this setting.^18,19^ Hypothalamic involvement with endocrine disturbance was not seen in a cohort of 131 patients with PCNSV,^18^ whereas endocrine findings were encountered in 10%-44% of CNS sarcoidosis patients.^14,20^ Optic nerve or chiasm involvement is rarely seen in PCNSV, and visual symptoms constitute mostly amaurosis fugax (1%), papilledema (5%) and visual field defect (21%);^19^ in neurosarcoidosis its frequency is up to 38%.^21,22^ Optic nerve and chiasm involvement are also uncommon in other RPD.^23^

Other neurological disorders, such as autoimmune inflammatory diseases (e.g. multiple sclerosis, systemic lupus erythematosus), infectious diseases (e.g. neuroborreliosis, neurolues, human immune deficiency, neurotuberculosis) and neoplasms are also possible differential diagnosis that should be – and in these cases were - excluded in a RPD with these additional findings.

Although it is often cited as a differential diagnosis, the exact frequency of neurosarcoidosis amongst RPD is unknown. A case series with 67 non-prion RPD patients had one patient with neurosarcoidosis, which accounted for 1.5% of the sample. Other publications, mostly case reports, emphasize RPD as a manifestation of neurosarcoidosis.^4-13^

Although uncommon, neurosarcoidosis is a possible diagnosis on patients with RPD. The diagnosis can be challenging when the disease is restricted to the nervous system. In these cases, histopathologic findings can be inconclusive, especially regarding the differential diagnosis of primary granulomatous CNS angiitis. Clinical presentation and imaging findings are the key to reaching a correct diagnosis in these patients.
